# Quantized response times are a signature of a neuronal bottleneck in decision

**DOI:** 10.3389/fncom.2014.00042

**Published:** 2014-04-11

**Authors:** Pietro Perona

**Affiliations:** Department of Electrical Engineering and of Computation and Neural Systems, California Institute of TechnologyPasadena, CA, USA

**Keywords:** decision, ideal observer model, poisson neurons, response time distributions, response time modeling, sequential probability ratio test, SPRT

## Abstract

The histograms of response times of optimal YES/NO decisions that are computed from a single sensory Poisson neuron are highly structured. In particular, response times in NO decisions are quantized to a small set of times, while response times in YES decisions have a multimodal structure. Both the times of NO decisions, as well as the modes of the the histogram of YES decisions, are associated to the number of action potentials that were necessary to reach the decision. Their value is a function of the firing rate of the neuron in response to the states of the stimulus.

## 1. Introduction

Consider an animal that is facing a binary decision (YES/NO, GO/NO-GO) in response to the presence/absence of a stimulus (light/no light, sound/no sound). The animal makes its decision based on the firing pattern of its sensory neurons. What is the best decision strategy, one that minimizes the response time while keeping the number of decision errors within an acceptable predefined limit? In a wide set of situations the best decision strategy is well known and it is based on Wald's sequential probability ratio test (SPRT) (Wald, 1945; Wald and Wolfowitz, 1948). Models of neuron-based decision that are based on SPRT have been successful at predicting the response time histograms of animals that are engaged in decision tasks, and neurophysiological correlates of key stages of the computation have been found (Gold and Shadlen, 2007), suggesting that the brain may be using this decision strategy. Here I explore the predictions of this theory when very few, one in the limit, neurons are engaged in the decision process. I find that response time histograms become highly structured, something that ought to be easy to reveal with simple behavioral/psychophysical experiments and might provide additional insight into how the brain computes decisions from sensory inputs.

In order to make this paper self-contained, in Section 2, I review SPRT and in Section 3, I review how to compute such decision based on the firing pattern of Poisson (or Bernoulli) neurons. In Section 4, I explore an example with physiologically plausible constants. In Section 5, I compute the properties of response time histograms for a single neuron. In Section I briefly discuss the case of multiple neurons involved in a decision. Section 8 describes the computational experiments. I conclude in Section 9 with a discussion of the main observations.

## 2. Optimal bayesian observer

I assume that the animal is sensing, and responding to, an underlying binary state of the world, e.g., the presence/absence of a given stimulus. I will call *C* = 0 one state (stimulus absence) and *C* = 1 the other state (stimulus presence). The animal's YES/NO decision is computed from the stimulus. The animal is rewarded if the response is appropriate, i.e., (*C* = 0) → NO and (*C* = 1) → YES, and the animal is not rewarded otherwise.

Call *X_t_* the measurements on which the decision is based. I will assume that *X_t_* is the firing pattern of one sensory neuron which will be firing vigorously if the stimulus is present, and will fire at a low level of activity if the target is absent. Here *t* is a discrete index of time, with observations starting at *t* = 1, thus, if *x_t_* is the piece of information that is acquired at time *t*, then *X_t_* = {*x*_1_, …, *x_t_*}. As I will explain below, the theory may be developed both in discrete time and in continuous time; the two formalisms yield identical results.

Wald's SPRT strategy works by repeating at each time *t* the following two steps:

First, compute the log ratio of the probability that *C* = 1 is true, vs the probability that *C* = 0 is true, given the available data *X_t_*:

(1) $$R_{t}=\log\frac{P(C=1|X_{t})}{P(C=0|X_{t})}$$

Second, compare *R_t_* to two thresholds, τ_0_ and τ_1_ (typically τ_0_ < 0 < τ_1_). If *R_t_* > τ_1_, then *C* = 1 is much more likely than *C* = 0, and decision YES is made. If instead *R_t_* < τ_0_, then decision NO is made. The third possibility is that τ_0_ < *R_t_* < τ_1_, i.e., the ratio is between thresholds. In this case no decision may be made because the information is insufficient, and one waits for the next piece of evidence *x*_*t* + 1_. The process is repeated until a decision is made.

In summary:

$$\begin{array}{l} \text{repeat for each }t\text{​​​​=1},\ldots \text{until decision reached}:\\ \text{obtain }x_{t},\text{compute }R_{t},\text{if}\{\begin{array}{l} R_{t}>\tau_{\text{1}}\;\to \text{decide YES}\\ R_{t}<\tau_{\text{0}}\;\to \text{decide NO}\\ R_{t}\in \left(\tau_{\text{0}},\tau_{\text{1}}\right)\to \text{wait for }x_{t\text{ + 1}} \end{array} \end{array}$$

Wald proved that this strategy is optimal (Wald, 1945; Wald and Wolfowitz, 1948), i.e., no strategy with the same error rate yields faster decisions. A review of this strategy in the context of neurophysiological models of decision may be found in Gold and Shadlen (2007).

The two thresholds τ_0_,τ_1_ determine the error rates, and may be computed from the expression for *R_t_* in Equation 1:

(2) $$R_{t}=\log\frac{P(C=1|X_{t})}{P(C=0|X_{t})}=\log\frac{P(C=1|X_{t})}{1-P(C=1|X_{t})}$$

where the denominator of the second fraction depends on the fact that *C* may only take two values, 0 and 1. Solving this expression for *P*(*C* = 1|*X_t_*) yields:

(3) $$P(C=1|X_{t})=\frac{1}{1+\exp(-R_{t}(X_{t}))}=g(R_{t})$$

**Figure 1:**
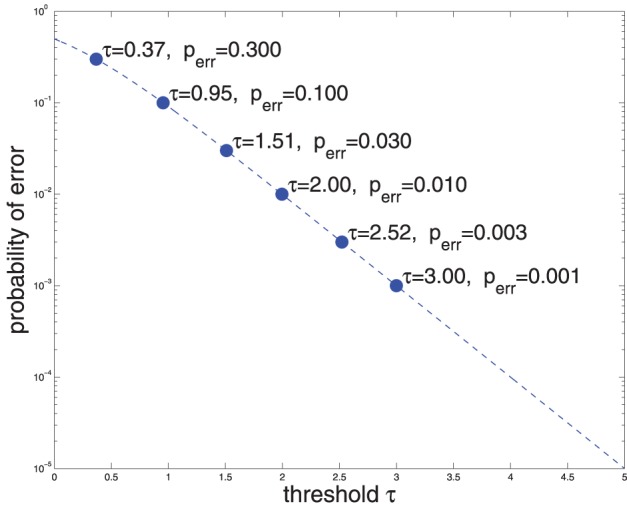
**Probability of error vs. threshold on the log-likelihood ratio *R_t_* (see Equations 2, 3)**.

Where *g*(·) denotes the logistic function. Thus, say, if *R_t_* = τ_1_ = 2, that means that *P*(*C* = 1|*X*) ≈ 0.99 (Equation 3 and Figure 1), i.e., the false accept error rate is 1%. Conversely, if a 1% false accept error rate is deemed acceptable, then (Equation 2) may be used to compute the upper threshold on *R_t_*: τ_1_ ≈ 2. Similarly, if a false reject rate of 1% is reasonable, then τ_0_ = −2 (for convenience of mental calculation I am using base 10 for both exp and log). Figure 1 shows the relationship of error probability and threshold magnitude.

Computing *R*_1_, *R*_2_, …, *R_t_* is easy from the class conditional probabilities *P*(*X_t_*|*C* = 1), *P*(*X_t_*|*C* = 0) if the measurements *x_t_* are independent when the state of the world *C* is known. Using Bayes' theorem:

(4) $$\begin{array}{l} R_{t}=\log\frac{P(C=1|x_{1},\ldots ,x_{t})}{P(C=0|x_{1},\ldots ,x_{t})}=\log\frac{P(C=1)}{P(C=0)}\\ \;+\sum_{s=1}^{t}\log\frac{P(x_{s}|C=1)}{P(x_{s}|C=0)}\\ \;=r_{0}+\sum_{s=1}^{t}r(x_{s})=R_{t-1}+r(x_{t}) \end{array}$$

where

$$\begin{array}{l} \;r_{0}=\log\frac{P(C=1)}{P(C=0)}\text{ (log ratio of priors)}\\ r(x_{t})=\log\frac{P(x_{t}|C=1)}{P(x_{t}|C=0)}\text{ (log ratio of probabilities}\\ \text{ given evidence }x_{t}) \end{array}$$

Equation 4 shows that the computation is a diffusion, i.e., it may be carried out recursively by updating the previous value *R*_*t*−1_ with a term *r*(*x_t_*) that depends only on the current observation *x_t_*, rather than on the whole set of observations *X_t_*. The fact that some binary decisions may be implemented by the brain with a diffusion was first suggested as a phenomenological model (Ratcliff and Hacker, 1981) and later shown to be optimal, under appropriate conditions (Gold and Shadlen, 2001).

## 3. Decisions based on one poisson neuron

As soon as one knows the statistics *P*(*X_t_*|*C*) of neuronal responses *X_t_* to stimuli *C* one is able to compute the log likelihood ratio *R_t_* explicitly. Here I will derive *R_t_* assuming that neurons produce patterns of action potentials that are distributed with Poisson statistics, a model that has been shown to be useful in many instances (Seung and Sompolinsky, 1993; Jazayeri and Movshon, 2006; Graf et al., 2011). More general Poisson-like models (Beck et al., 2008) may be used in the following analysis. They have the disadvantage of being more complex to implement and analyze and therefore I will confine myself to Poisson here.

**Figure 2:**
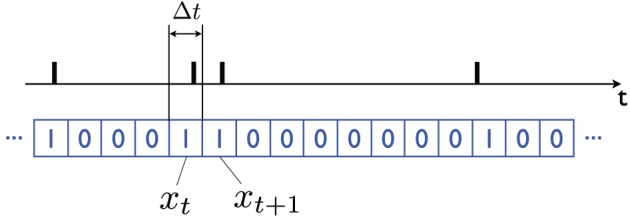
**Bernoulli representation of a sequence of action potentials**. In the black plot (above) the action potentials produced by a neuron over time are indicated with vertical bars. The binary *x_t_* ∈ {0, 1} variable (blue plot, below) is obtained by dividing time into (short) bins of length Δ*t*; *x_t_* = 1 if an action potential is observed in the corresponding time interval *t*, and it is equal to 0 otherwise. The bins should be short enough that the probability of observing multiple action potentials in one bin is very small (e.g., Δ*t* = 1 ms). By taking the limit Δ*t* → 0 one obtains a Poisson model of neuronal firing.

The equations are particularly simple if one uses a Bernoulli approximation to the Poisson distribution: call *X_t_* the firing pattern of a neuron. I will assume that time has been discretized in small non-overlapping identical bins, e.g., time bins that are Δ*t* = 1 ms long, and that the random variable *x_t_* ∈ {0, 1} represents whether an action potential is observed during the interval corresponding to time-bin *t*, in which case *x_t_* = 1, otherwise *x_t_* = 0 (see Figure 2).

Consider a neuron whose expected firing rate is λ_0_ action potentials per second when *C* = 0, and λ_1_ action potentials per second when *C* = 1 (if one models the firing rate with a Poisson process, λ is the expectation). In this case one may compute the value of *r*(*x_t_*) to be used in the diffusion Equation 4:

$$r(x_{t})=\log\frac{P(x_{t}|C=1)}{P(x_{t}|C=0)}\;$$

the variable *x_t_* takes only two values, 0 and 1. Thus:

(5) $$\begin{array}{l} r(0)=\log\frac{1-p_{1}}{1-p_{0}}\\ r(1)=\log\frac{p_{1}}{p_{0}} \end{array}$$

where *p*_0_ and *p*_1_ are the probabilities of detecting an action potential in a given time bin when the world is in state *C* = 0 and *C* = 1 respectively. Thus, indicating with Δ*t* the duration of a time bin, chosen so that λΔ*t* «1:

$$\begin{array}{l} p_{0}\approx \lambda_{0}\Delta t\\ p_{1}\approx \lambda_{1}\Delta t \end{array}$$

The limit for Δ*t* → 0 yields the continuous model. There is only one delicate point: in the limit *r*(0) → 0; however, δ = *r*(0)/Δ*t*, the linear drift rate of the diffusion when no action potentials are observed, is different from zero. Thus:

(6) $$\begin{array}{l} \delta =\underset{\Delta t\to 0}{\lim}\frac{r(0)}{\Delta t}=\underset{\Delta t\to 0}{\lim}\frac{1}{\Delta t}\log\frac{1-p_{1}}{1-p_{0}}\\ \;=\underset{\Delta t\to 0}{\lim}\frac{1}{\Delta t}\log\frac{1-\lambda_{1}\Delta t}{1-\lambda_{0}\Delta t}=\log(e)(\lambda_{0}-\lambda_{1}) \end{array}$$

(7) $$\begin{array}{l} r(1)=\log\frac{p_{1}}{p_{0}}=\log\frac{\lambda_{1}}{\lambda_{0}}(\text{independent of }\Delta t,\text{thus the limit}\\ \text{ is trivial)} \end{array}$$

By taking the limit for Δ*t* → 0 we obtained the exact equations for the Poisson model (Jazayeri and Movshon, 2006; Chen et al., 2011). In Equation 6 the limit may be computed by considering the Taylor expansion log(1 + *x*)log(*e*) = *x* + *O*(*x*^2^), where *O*(*x*^2^) indicates higher order terms that vanish when one takes the limit. When using base 10 for the log, as in Table 1 and in Section 4, then log_10_(*e*) ≈ 0.4343. When using natural logs, then, of course, log(*e*) = 1.

**Table 1:** **Parameters used in the example of section 4 and in the simulations shown in Figures 4, 5. For convenience, base 10 was used for both exp and log**.

**Independent variables:**	
λ_0_ = 1 spike/s	Firing frequency of neuron when *C* = 0
λ_1_ = 10 spikes/s	Firing frequency of neuron when *C* = 1
*P*(*C* = 0) = 0.5	Prior probability of *C* = 0
*P*(*C* = 1) = 0.5	Prior probability of *C* = 1
**Derived variables:**	
*r*_0_ = log(*P*(*C* = 1)/*P*(*C* = 0)) = 0	Initial value *R*_0_ of the diffusion (Equation 4)
*r*(1) = log(λ_1_/λ_0_) = 1	Diffusion increment, action potential at time *t* (Equation 7)
δ = log_10_(*e*) (λ_0_ − λ_1_) ≈ −3.9 s^−1^	Diffusion drift when no action potential is observed (Equation 6)

**Figure 3:**
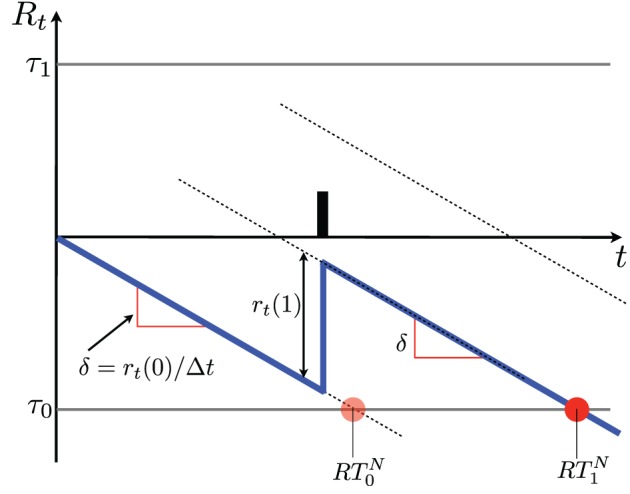
**Schematics of a diffusion resulting from SPRT**. The vertical axis indicates the log probability ratio *R_t_*. The diffusion is indicated in blue. In the absence of action potentials the diffusion drifts by δ units/s and reaches the lower threshold at time *RT^N^*_0_ (see section 5 and Equation 8), where a NO decision is made. When an action potential occurs the diffusion jumps by *r*(1). When one of the two thresholds τ is crossed, a decision is made. In this case the decision is NO; it is indicated by a red dot. It is made at time *RT^N^*_1_ (see Equation 8). Notice that if the time of the action potential changes within the interval *t* ∈ (0, *RT^N^*_0_), the decision is made at the same time. A number of diffusions produced by a simulation with the parameters shown in Table 1 are shown in Figures 4, 5.

According to Equations 6, 7 the diffusion will mostly drift linearly at a rate δ, and present jumps of height *r*(1) whenever an action potential is observed (see Figure 3).

Notice that if one assumes, without loss of generality, that λ_1_ > λ_0_, then the diffusion jumps will always be positive, i.e., upwards and the drift will always be negative, i.e., downwards. i.e., action potentials always contribute evidence toward *C* = 1, while quiet periods always contribute evidence toward *C* = 0.

## 4. A concrete example

**Figure 4:**
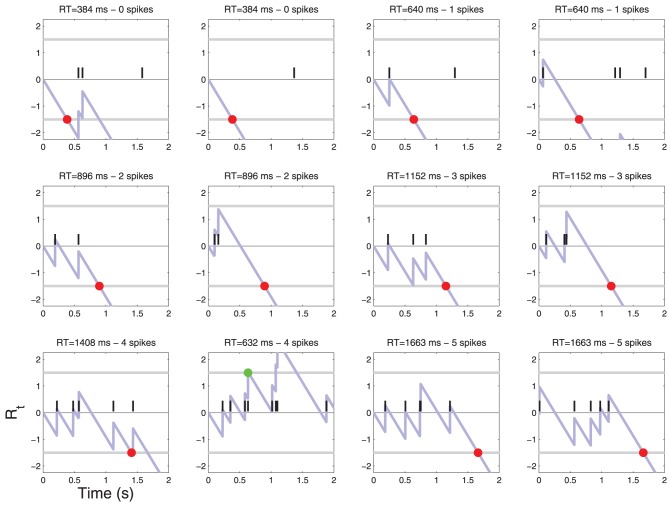
**Patterns of action potentials and ensuing decisions when *C* = 0**. Action potentials are represented by black vertical bars along the time axis. The corresponding diffusion *R_t_*(*X*) (Equation 4) is represented by a light blue line. When *R_t_* crosses one of the two thresholds τ a decision is made. YES decisions are represented by green dots and NO decisions by red dots. One may notice one false alarm error in the last row. The parameters of the simulation are shown in Table 1. For convenience, examples are sorted by the number of action potentials that were sufficient to reach a decision. For a frequency of each such event see Figure 6.

**Figure 5:**
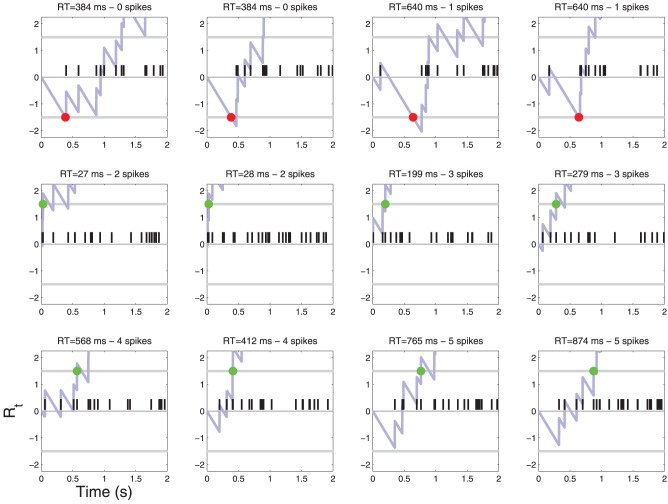
**Patterns of action potentials and ensuing decisions when *C* = 1**. Same parameters as in Figure 4. One may notice false reject errors in the first row.

In order to develop one's intuition it is useful to explore a concrete case. Suppose that a neuron responds to stimuli with parameters indicated in Table 1, which correspond to a neuron firing briskly at 10 Hz when the stimulus is present (*C* = 1) and at some “resting” level of 1 Hz when the stimulus is absent (*C* = 0). The diffusion starts at a value of *R*_0_ = 0, it is incremented by *r*(1) = 1 units whenever an action potential is observed (this is a rare event) and it drifts by δ = −9 log_10_(*e*) ≈ − 3.9 units/s during the intervals where no action potentials are observed (see Figure 3). Simulations of this are shown in Figures 4, 5.

Simple calculations allow one to estimate the expected response time for decisions when *C* = 1 and when *C* = 0.

Suppose that we consider an error rate in the neighborhood of 3% acceptable, then we should use thresholds τ_1_ = 1.5 and τ_0_ = −1.5 (Equation 3, Figure 1). Since one action potential increases the diffusion value by one unit, and 1 < τ_1_, it will take at least two action potentials to reach a YES decision, while a sufficiently long time interval with no action potentials will lead to a NO decision (see Figure 3). We explore in the following the response time for YES and NO decisions when *C* = 1 and when *C* = 0. A more systematic analysis is presented in Section 5.

**YES decision after 2 action potentials** (*C* = 1)—Let's consider first the case where the decision is made after two action potentials. After two action potentials the value of the diffusion is *R_t_* = 2 + δ*t* (*t* is the time that has passed since the beginning of observation). Thus a YES decision may be taken only if *t*_2_ < (2 − τ_1_)/|δ|≈ 128 ms. The neuron fires with frequency λ_1_ = 10 spikes/s, which makes it somewhat unlikely that two action potentials will be observed in 128 ms (the probability is about 0.23, which may be computed considering the Poisson distribution with expectation λ = *t*_2_ λ_1_). Thus, YES decisions that are based on two action potentials will only happen in a minority of cases.

**YES decision after 3 action potentials** (*C* = 1)—Consider now the case where the YES decision is made after three action potentials. This decision may not be made when *t* < 128 ms since in that case it would be made after the first two action potentials (see previous paragraph). Thus the decision time is at least 128 ms. Furthermore, following the reasoning in the previous paragraph, the decision time will at most be *t*_3_ = (3 − τ_1_)/|δ|≈ 384 ms. The probability that at least three action potentials are observed in less than 384 ms is about 0.47.

**NO decisions** (*C* = 0)—Now consider the *C* = 0 case. If no action potentials are observed it will take τ_0_/δ ≈ 1.5/3.9 ≈ 0.384 ms for a negative decision to be made. However, if one action potential is observed during this time, then the diffusion will be incremented by 1 and one will have to wait ≈ 1/3.9 ≈ 0.256 s longer for a negative decision. When *C* = 0 it is quite likely that no action potentials are observed during a 0.384 s time interval since λ_0_ = 1 s/s; the probability of this event is ≈ 0.68 (computed from a Poisson distribution *P*(*k* = 0; λ) with λ = 0.384). Thus, one would expect the majority of negative decisions to be made after no action potentials are observed and a time of about 0.384 s has elapsed.

**Figure 6:**
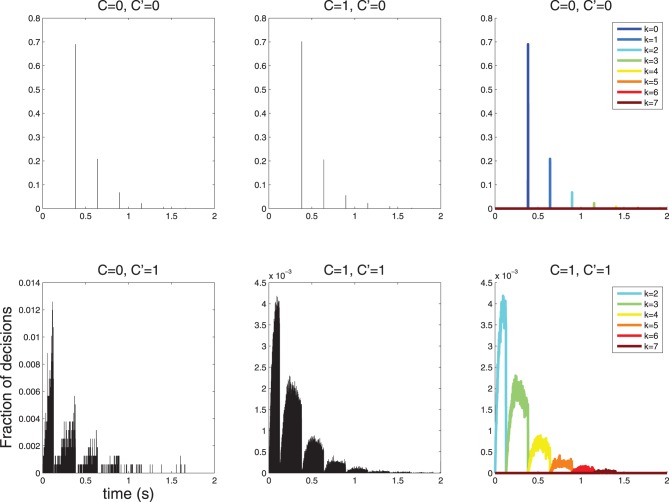
**Histograms of 2 · 10^5^ decision times for diffusions with parameters as in Table 1 and Figures 4, 5**. The symbol *C* indicates the stimulus and the symbol *C*′ indicates the decision; thus, the combination *C* = 0, *C*′ = 1 denotes false alarms and *C* = 1, *C*′ = 0 indicates false rejects. (**Top-Left, Top-Center**) The histogram of response times is sparse for *C*′ = 0, in line with the theoretical prediction (Section 5). In this case, decisions are more likely to be made with 0 action potentials, slightly less likely with 1, then 2 etc. (**Bottom-left**, **Bottom-Center**) The histograms for *C*′ = 1 is not sparse; however, it is lumpy. (**Top-Right**, **Bottom-Right**) The structure of the *C*′ = 0 and *C*′ = 1 histograms is clear when separate histograms are drawn for decisions made after *k* = 0,…,6 action potentials. Notice that for *C*′ = 1 no such decisions involve fewer than 2 action potential, since 1 action potential is insufficient to cross the τ_1_ = 1.5 threshold as discussed in Section 4.

I simulated such a process and obtained histograms of decision times (Figure 6). The histogram of decision times for *C* = 0 is sparse. Only a few discrete decision times are observed. This issue is explored in Section 5.

## 5. Sparse RT histograms

Each decision is made once a small number of action potentials has been observed. Without loss of generality, let's assume that the neuron fires more vigorously when *C* = 1 than when *C* = 0, i.e., λ_1_ > λ_0_ ⇒ *p*_1_ > *p*_0_. Then *r*(1) > 0 > δ (see Equations 6, 7).

**NO decisions—**Since δ < 0, decisions made after observing *k* = 0 action potentials must be of type NO and are made in exactly *RT^N^*_0_ = τ_0_/δ s (Figure 3). For *k* = 1 a NO decision takes longer because the diffusion is incremented by *r*(1) when the action potential is observed (Figure 3); therefore, it takes −*r*(1)/δ longer to reach the threshold, thus *RT^N^*_1_ = *RT^N^*_0_ − *r*(1)/δ. The general expression for the NO decision time after *k* action potentials is therefore:

(8) $$RT_{k}^{N}=\frac{\tau_{0}}{\delta }-k\frac{r(1)}{\delta }$$

Therefore, one would predict that when only one neuron is involved, decision times are sparse and no other decision time may be observed.

Using the constants of Table 1 as in the example above yields: *RT^N^_k_* = (1.5 − *k*)/(9 log_10_(*e*))), i.e., one would predict the following discrete decision times 384, 640, 895, 1151 ms etc. for *k* = 0, 1, 2, 3, …. This is precisely what is observed in the simulation shown in Figure 6 (left).

**YES decisions—**As shown in Figure 6, the histogram of response times is lumpy. It is possible to predict this observation, and to see that each mode of the histogram corresponds to a different number *k* of action potentials. The time it takes to compute a YES decision that is based on *k* action potentials has a lower and an upper bound. Let's call the bounds *RT*^*Y, l*^_*k*_ and *RT^Y, u^_k_*. The maximum time that it may take for a YES decision to be made is easy to compute: *k* action potentials increase the diffusion by *kr*(1) units. To obtain a YES decision after *k* action potentials in the amount of time *RT^Y, u^_k_* the diffusion will reach a value equal to τ_1_ after *RT^Y, u^_k_* seconds, which implies a downwards drift of δ *RT^Y, u^_k_* = *kr*(1) − τ_1_ units. Solving for the upper bound yields:

(9) $$RT_{k}^{Y,\;u}=\frac{\tau_{1}-k\cdot r(1)}{\delta }$$

The lower bound may also be computed considering the fact that it is achieved when a decision based on *k* − 1 action potentials is missed by a hair's breath and is followed immediately by another action potential, which overshoots the threshold. Thus:

(10) $$RT_{k}^{Y,l}=\frac{\tau_{1}-(k-1)\cdot r(1)}{\delta }$$

Using the constants of Table 1 yields the following predictions: *RT*^*Y*, l^_2_ = 0 s, *RT*^*Y, u*^_2_ = 0.128 s, *RT*^*Y*, l^_3_ = 0.128 s, *RT*^*Y, u*^_3_ = 0.384 s, *RT*^*Y*, l^_3_ = 0.384, *RT*^*Y, u*^_3_ = 0.640 s etc. The simulation shown in Figure 6 (right) are consistent with these predictions.

If one compares equations 8 with 9 one can readily see that when τ_1_ = − τ_0_ the sparse RT for NO decisions and the “zeros” of the RT histogram of YES decisions are the same, i.e., YES decisions mostly happen at times that are different from NO decisions.

**Figure 7:**
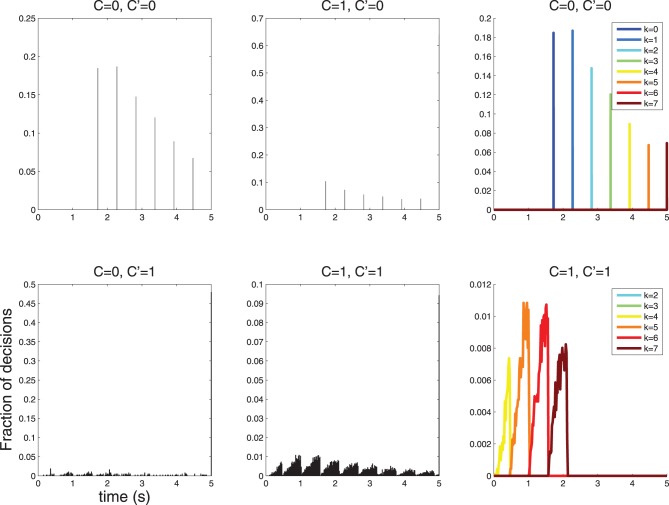
**When λ_1_ is reduced the spacing of the peaks in the response time histograms becomes larger**. Decisions take longer, since the neuron fires less vigorously, and firing rates between *C* = 0 and *C* = 1 are more similar. Here λ_0_ = 1, λ_1_ = 3; compare with Figure 6 where λ_0_ = 1, λ_1_ = 10.

One last question one may ask is how the various parameters (τ and λ) affect the spacing between the response times, and the height of the peaks, in the NO decision sparse histogram. It is intuitive that the larger the spacing and the higher the second peak w.r. to the first one, the easier it will be to observe the sparse nature of the response time histogram in an *in-vivo* experiment where multiple sources of noise and variability will tend to blur away the sparse/lumpy nature of the histograms. From Equation 8 the spacing between peaks is equal to:

(11) $$S=\frac{r(1)}{\delta }=\frac{\log(\lambda_{1})-\log(\lambda_{0})}{\log(e)(\lambda_{1}-\lambda_{0})}$$

therefore the spacing is maximum for λ_1_ → λ_0_. An experiment where *C* = 0 does not stimulate the neuron, and *C* = 1 stimulates the neuron lightly may therefore be the best option for revealing sparse response time histograms (see Figure 7).

## 6. Decisions involving multiple neurons

Decisions involving multiple neurons firing independently may be made using SPRT and Equation 4 (detailed equations for Poisson neurons may be found in Chen et al., 2011). Each action potential contributes to the diffusion independently of the other action potentials, and the contribution of each action potential is *r^i^*(1) = log(λ^*i*^_1_) − log(λ^*i*^_0_) (same as the case for a single neuron, see Equation 7), where *i* is the index of the neuron that generated the action potential. Thus, neurons that respond very differently to *C* = 0 and *C* = 1 (and thus λ^*i*^_0_ and λ^*i*^_1_ are very different) will contribute strongly to the diffusion, while neurons for which λ^*i*^_0_ ≈ λ^*i*^_1_ will have little influence.

If the population of neurons responds overall asymmetrically to *C*, then one would still expect to observe lumpy response time histograms if the neurons are not too many. In the special case where λ^*i*^_1_ > λ^*i*^_0_ ∀ i (e.g., when *C* = 1 corresponds to a sound and *C* = 0 corresponds to no sound, and all neurons are excited by sound), then all action potentials will send the diffusion upwards and the only way a NO decision may be taken is by the diffusion drifting toward the negative threshold. In this case the NO response time histogram will be sparse. However, the analysis of this case becomes considerably more tedious than the analysis presented in Section 5.

**Figure 8:**
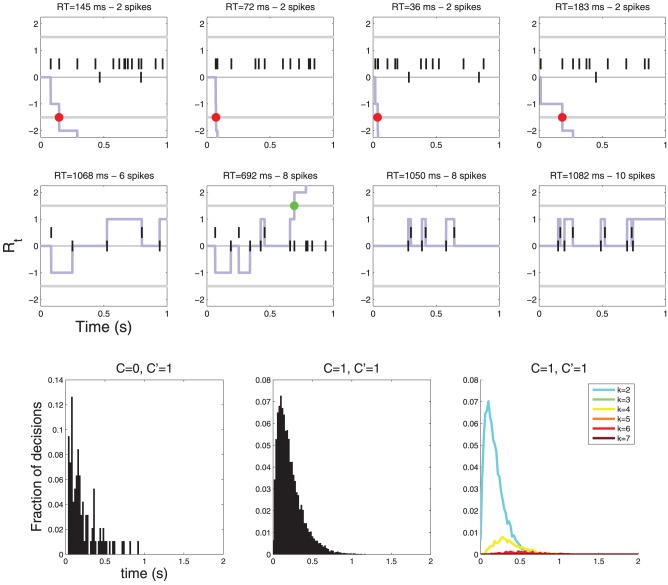
**Diffusions for a decision involving two neurons**. The neurons are symmetric with respect to *C*: one fires more vigorously for *C* = 0 (λ_0_ = 10, λ_1_ = 1) and the other for *C* = 1 (λ_0_ = 1, λ_1_ = 10). In this case there is no drift (the two drift terms cancel) and the diffusion only moves when an action potential is observed. (**Top row**) examples of diffusions where a decision is reached quickly, (**Middle row**) examples of diffusions where decisions are reached after multiple action potentials. YES decisions are represented by green dots and NO decisions by red dots. (**Bottom row**) Histograms of response times. Notice that the response time histograms are neither sparse nor lumpy (compare with Figure 6).

If, on the other hand, the neurons are symmetrical w.r. to *C*, i.e., some neurons respond preferentially to *C* = 0 and some to *C* = 1, then the drift term is zero because drift terms for different neurons are equal and opposite and thus cancel each other. In this case the response time histograms are neither lumpy, nor sparse (see Figure 8).

## 7. Firing rates and response times

**Figure 9:**
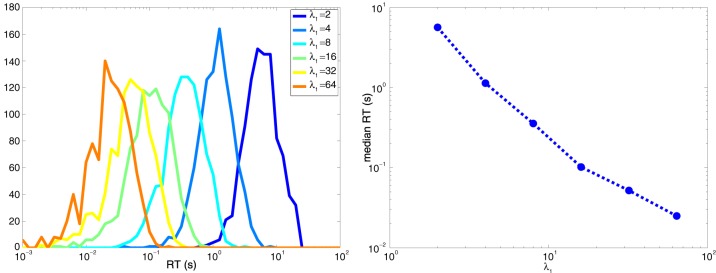
**Decision times are shorter when λ_max_ >> λ_min_. (Left)** Histogram of the response times for the two-neuron system explored in Figure 8. (**Right**) Median response time as a function of λ_max_ when λ_min_ = 1 spike per second.

It is intuitive that response times will be lower when the maximum firing rate λ_1_ is much larger than the minimum firing rate λ_0_. Consider Equation 8. Decisions based on zero action potentials take τ_0_/δ = τ_0_/(λ_0_ − λ_1_). Since τ_0_ is negative, these decisions are quicker when λ_1_ >> λ_0_, which makes the magnitude of the denominator larger. Similarly, when *k*>0, decisions take are quicker when λ_1_ >> λ_0_ since the numerator increases logarithmically, while the denominator increases linearly. Figure 9 shows the behavior of RT vs λ_max_ when λ_min_ = 1 for the symmetric case of two neurons described in Section6, where for one neuron λ_1_ = λ_max_ and λ_0_ = λ_min_ and vice-versa for the other neuron.

## 8. Methods

The optimal Bayesian observer based on SPRT was implemented in Matlab using the Bernoulli method described in Section 3 with Δ*t* = 1 ms and other parameters as shown in Table 1, unless otherwise specified in the text.

## 9. Discussion and conclusions

The analysis I presented predicts that when binary decisions are computed by a mechanism involving a single neuron, one will observe sparse response time histograms for NO responses, and lumpy response time histograms for YES responses.

A number of additional observations are possible:

1. The histograms will still look highly structured when few neurons are involved, provided that the YES/NO decision is perceptually asymmetric (e.g., detecting the presence vs the absence of a sound, a vibration, or a light spot).
2. The response time histogram will look like a log-normal distribution when the neurons involved respond symmetrically to *C*.
3. Even if the timing of the action potentials produced by the input neuron(s) are unpredictable and uncorrelated, as modeled by Poisson statistics, the timing of action potentials produced by the neurons that compute a decision is highly structured; it is tightly quantized in the case of NO decisions. Conceivably, this fact will enable additional computations where synchronization between action potentials is required.
4. If quantized/structured response time histograms are observed in an experiment, it may be possible not only to predict that a small number of neurons is involved, but also to estimate their firing rate.
5. The predictions I make are not affected by the *real* statistics of the neuron's response. They are a consequence of using SPRT and the *assumption* that the neuron's statistics is Poisson. It is impossible to decide whether a short sequence of action potentials is governed by Poisson or other statistics. I suspect that any Poisson-like assumption will lead to the same qualitative prediction.

Observing sparse response time histograms experimentally is difficult for a number of reasons:

1. In most systems the number of neurons involved in the computation is more than a handful.
2. If the task is symmetric (e.g., discriminate between a red and a green light), then there will be neurons tuned to both conditions making both YES and NO histograms continuous, rather than sparse.
3. Motor response and neural propagation delays are themselves a random variable which, if summed to the perceptual response time, will blur away the sparse nature of the NO histograms.

I believe that one should be able to design single-neuron asymmetric preparations. For example, by stimulating a single ganglion cell in the retina with a small light dot, or stimulating a single tactile receptor in the skin of the back, where receptors are sparse. If such experiments prove to be possible, response time histograms would provide a wealth of information on the mechanisms involved in decision, including the firing rate of the neuron involved.

### Conflict of interest statement

The authors declare that the research was conducted in the absence of any commercial or financial relationships that could be construed as a potential conflict of interest.
